# Bone turnover markers in HIV-infected women on tenofovir-based antiretroviral therapy

**DOI:** 10.4102/sajhivmed.v18i1.739

**Published:** 2017-12-06

**Authors:** Mwila Mulubwa, Michelle Viljoen, Iolanthe M. Kruger, Herculina S. Kruger, Malie Rheeders

**Affiliations:** 1 Centre of Excellence for Pharmaceutical Sciences (Pharmacen), Division of Pharmacology, North-West University, South Africa; 2 Department of Pharmacology and Clinical Pharmacy, School of Pharmacy, Faculty of Natural Sciences, University of the Western Cape, South Africa; 3 Africa Unit for Transdisciplinary Health Research (AUTHeR), North-West University, South Africa; 4 Centre of Excellence for Nutrition (CEN), North-West University, South Africa; 5 Medical Research Council Hypertension and Cardiovascular Disease Research Unit, North-West University, South Africa

## Abstract

**Background:**

Tenofovir disoproxil fumarate (TDF) antiretroviral therapy is associated with disruption of the bone turnover process.

**Objectives:**

The objective of this study was to determine the association between tenofovir (TFV) plasma concentration and various bone turnover markers and compare these markers in HIV-infected women and HIV-uninfected controls.

**Method:**

A cross-sectional sub-study included 30 HIV-infected women on TDF and 30 HIV-uninfected matched participants. Serum calcium (SrCa), serum phosphate (SrP), C-terminal telopeptide (CTx), parathyroid hormone (PTH), alkaline phosphatase (ALP), C-reactive protein (CRP), vitamin D (VitD) and bone mineral density (BMD) were measured. Plasma TFV was assayed on HPLC-MS/MS. The statistical tests applied were Mann–Whitney test, unpaired *t*-test, analysis of covariance, regression and correlation analysis.

**Results:**

In HIV-infected women, no correlation existed between plasma TFV concentration and CTx, PTH, ALP, SrCa, SrP, VitD or BMD (*p* > 0.05). After adjusting for smoking and alcohol use, ALP (*p* < 0.001), CTx (*p* = 0.027) and PTH (*p* = 0.050) were significantly higher in HIV-infected compared to HIV-uninfected women. Women with TFV concentration ≥ 120 ng/mL had higher PTH concentrations (*p* = 0.037) compared to those with ≤ 100 ng/mL. Significant correlations between SrCa and PTH and SrCa and SrP including CTx and PTH (*p* < 0.05) were present in HIV-uninfected women while absent in HIV-infected counterparts (*p* > 0.05).

**Conclusion:**

The results indicate possible increased bone turnover at higher TFV concentrations. The normal regular bone turnover processes in HIV-infected women on TDF therapy are altered. Larger studies are warranted to confirm these results.

## Introduction

Low bone mineral density (BMD) is common among persons infected with human immunodeficiency virus (HIV).^1^ In HIV-infected people, causes of osteoporosis, fractures and low BMD are multifactorial, involving traditional risk factors (smoking, alcohol use, low body weight and physical inactivity), but also genetics and HIV-infection *per se*.^1,2^ Exposure to antiretroviral treatment (ART), especially nucleoside reverse transcriptase inhibitors (NRTIs) and protease inhibitors (PIs) in HIV-infected people, is associated with low BMD.^3^ Osteomalacia was reported in patients on a tenofovir disoproxil fumarate (TDF)-based ART regimen that was characteristic of hypophosphataemia and increased serum alkaline phosphatase (ALP).^4^

In clinical studies, TDF-based ART regimens were reported to be associated with decreased BMD, characterised by increased serum concentrations of bone turnover markers (BTMs). These include bone resorption markers such as C-terminal telopeptide (CTx) and bone formation markers such as procollagen type 1 N-terminal propeptide (P1NP), bone-specific ALP (BALP) osteocalcin and ALP.^5,6,7,8^ Furthermore, increased bone turnover, after initiation of TDF therapy, was characterised by elevated serum concentrations of parathyroid hormone (PTH).^9^ However, elevated serum concentrations of PTH were more profound in patients with vitamin D (VitD) deficiency.^10,11,12^

Tenofovir disoproxil fumarate undergoes initial di-ester hydrolysis in plasma to tenofovir (TFV). A proposed bone loss mechanism associated with TFV, a phosphonate, is selective uptake by osteoclasts via a mechanism similar to that of bisphosphonates, which eventually cause cellular stress.^13^ Resulting cellular stress perturbs deoxyribonucleic (DNA) synthesis by altering gene expression that is involved in signalling osteoblast activity resulting in decreased bone formation.^13,14^ Despite studies^3,5,7^ performed on the relationship between TDF-use and bone turnover, few studies have related actual measured plasma TFV concentrations to BTM and this information is lacking in South Africa. In one study, higher serum calcium (SrCa) and 25-dihydroxyvitamin D were found in patients who had TFV plasma concentration above 95.3 ng/mL, while free 1,25-dihydroxyvitamin D was lower.^15^ The use of TFV plasma concentrations to correlate with BTM provides better evidence for the physiological association between TFV and BTM than mere ‘TDF-use’ status. The advantage of measuring TFV plasma concentrations is the ability to determine and set ranges of TFV plasma concentrations that correspond with several range values of BTM.

The objectives of this study were to determine the relationship between plasma TFV concentration, BTM and BMD in HIV-infected women on TDF-based ART and to compare this with the matched HIV-uninfected control group.

## Materials and methods

### Study design and participants

This pilot cross-sectional sub-study forms part of the South African arm of the global Prospective Urban and Rural Epidemiology (PURE-SA) study and has been described in detail elsewhere.^16^ Cross-sectional data were collected from 462 black women with similar socio-demographic characteristics, mainly from the low-income group. Data collection took place between October 2012 and August 2013. For the purpose of this study, 60 women (*n* = 30 HIV-infected and *n* = 30 non-infected), aged between 37 and 87 years, were included. HIV-infected women on TDF-based ART reported to be taking 300 mg TDF *nocte* were matched with an HIV-uninfected female group according to their age and body mass index (BMI).

Participants were fully informed about the outcomes of the study. Each participant was required to sign a written informed consent before participating in within the PURE-SA study (ethical approval number: NWU-00016-10-A1). The Research Ethics Committee of the North-West University and North West Department of Health approved this sub-study. Participation was entirely voluntary and women could withdraw from the study at any time without any consequences. On the day of data collection, participants were transported to the North-West University Metabolic Clinic in Tlokwe (urban site) and at Sethlare Lodge in Ganyesa (rural site) on pre-arranged dates. Qualified pharmacists and assistants recorded medication history on a structured questionnaire. The study was performed according to the principles outlined in the Declaration of Helsinki as revised in 2013.

Participants with medical history of osteoporosis or taking osteoporosis preventing medication were excluded from this sub-study.

### HIV testing

Written informed consent was obtained from each participant after pre-counselling before participation. Participants’ HIV status was determined using the First Response (Premier Medical Corporation, Kachigam, India) rapid HIV card test using whole blood. This test was performed according to the protocol of the National Department of Health of South Africa. If the First Response test was positive, it was confirmed with the Pareeshak card test (BHAT Bio-Tech India, Bangalore, India). Feedback on results was given by two trained counsellors during individual sessions just before the participants were transported back to their homes. Infected participants were referred to their local clinic or hospital for follow-up and CD4 cell counts and treatment advice.

### Biochemical analyses

Blood samples for all participants in fasting state were collected in the morning between 08:00 and 10:00 by a registered research nurse. Plasma and serum samples were prepared according to standard methods and stored at −80 °C until analyses were performed. CTx, PTH and total VitD (25-hydroxyvitamin D) were analysed using the Roche Elecsys 2010 COBAS system (Roche Diagnostics, Indianapolis, IN, USA). The ALP, serum phosphate (SrP), SrCa and C-reactive protein (CRP) were analysed on a Cobas Intergra 400 plus (Roche, Forrenstrasse Switzerland). The data on serum creatinine, estimated creatinine clearance and estimated glomerular filtration rate in both HIV-infected and uninfected participants have been described in detail elsewhere.^17^

### Plasma tenofovir analyses

Plasma TFV was quantified by a validated high-performance liquid chromatography method^18^ with expected range of 75 ng/mL–148 ng/mL which was observed in patients with normal renal function.^19^ The lower and upper limit of quantification was 12.5 ng/mL and 600 ng/mL, respectively. The mean linear regression coefficient (*r^2^*) of the calibration curve was 0.9958 with nine calibration points.^18^ Inter- and intra-day precision and accuracy was in the range < 15% of the relative standard error and mean recovery was 96.9%.^18^

### Questionnaires

A semi-structured questionnaire was used to obtain demographic information, as well as dichotomous information (yes or no) on the use of tobacco and alcohol.

Baseline information on the duration of TDF-based ART and non-TDF regimens was obtained from clinic and hospital files retrospectively from the facility where participants received health care services.

### Bone mineral density assessment

Bone mineral density was assessed at distal and ultra-distal sites of the forearm (non-dominant arm) using a DTX-200 peripheral DXA system (Osteometer MediTech, Hawthorn, California, USA).^20^ BMD was expressed as absolute amount (g/cm^2^) of bone mass per unit area (areal density). A qualified radiographer performed all measurements on the same Osteometer.

### Anthropometric measurements

Anthropometrical measurements included weight and height and were measured using standardised methods, with calibrated instruments by an International Society of the Advancement of Kinanthropometry–accredited anthropometrist (Precision Health Scale, A & D Company, Japan; Leicester Height Measure, Seca, Birmingham, UK).

### Statistical analyses

The HIV-uninfected control group was selected using propensity score matching with the HIV-infected women. Age and BMI were used as predictors to model case or control membership in a multivariable logistic regression model with optimal match tolerance of 0.26. Sampling of controls was carried out without replacement.

Variables in both groups of women were tested for normality using Shapiro–Wilk test and visual inspection of their respective normal Q–Q plots. Unpaired *t*-test was used to compare variables between the HIV-infected and uninfected groups if data were normally distributed. Results were presented as mean and standard deviation (SD) in the tables. Mann–Whitney test was performed if data were skewed and results were presented as median and interquartile range (IQR).

Adjusted analyses for smoking status and alcohol use were performed using analysis of covariance (ANCOVA). Tests for associations were performed using linear regression between BTM or BMD (dependent variable) and HIV-infected status (TDF exposure) or uninfected status, controlling for smoking and alcohol use. Pearson correlations were performed between plasma TFV concentration, BTM and BMD in HIV-infected women.

Correlations among BTM were performed conditioned by TFV concentration in order to explore patterns of relationships among BTM and assessed whether TFV concentration affected the correlations (physiological relationship) among BTM. The upper value of TFV concentration range at which some significant correlations among BTM occurred was taken as the cut-off point. Therefore, comparisons of mean or median values of BTM and CRP between two groups were defined by the cut-off point.

Pearson correlations between SrCa and PTH, SrCa and SrP as well as CTx and PTH were also performed in HIV-infected and uninfected women.

A two-tailed significance testing level of *p* < 0.05 was used and statistical analyses were performed using IBM^®^ SPSS^®^ Statistics software, version 22.

#### Ethical consideration

Each participant was required to provide written informed consent before taking part in the PURE-SA study. The Research Ethics Committee of the North-West University (NWU-00016-10-A1) and North West Department of Health approved this sub-study.

## Results

Participants’ demographic information and bone variables are presented in Table 1. The HIV-uninfected group was significantly older than the HIV-infected group (*p* = 0.008) but age was already adjusted for when selecting controls via propensity score matching. ALP (112 ± 27.7 U/L vs. 78.7 ± 25 U/L; *p* < 0.001), CTx (0.68 ± 0.4 ng/mL vs. 0.56 ± 0.4 ng/mL; *p* = 0.027) and PTH (56.30 ± 32 pg/mL vs. 46.3 ± 18 pg/mL; *p* = 0.05) were higher in HIV-infected women compared to their matched HIV-uninfected counterparts. No significantly different VitD concentrations were observed between the two groups even after adjusting for seasonal variation (data not shown).

**TABLE 1:** Demographic characteristics, bone turnover markers and bone mineral density of HIV-infected women compared to the uninfected control group.

Variable (Mean and SD)	HIV-infected (*n* = 30)	HIV-uninfected (*n* = 30)	*p* †	Adjusted *p*‡
Age (years)	53 ± 9.3	60 ± 11	0.008§*	-
BMI (kg/m^2^)	24.2 ± 9.5	26.2 ± 8.2	0.333	-
Weight (kg)	58 ± 22.5	64.7 ± 20.5	0.179	-
TDF ART exposure (months)	16.2 ± 8.6¶	-	-	-
HIV disease duration (months)	37.8 ± 24.7††	-	-	-
SrCa (mmol/L)	2.08 ± 0.2	2.12 ± 0.2	0.406	0.835
SrP (mmol/L)	1.05 ± 0.2	0.99 ± 0.2	0.204	0.165
CTx (ng/mL)	0.68 ± 0.4	0.56 ± 0.4	0.181	0.027*
PTH (pg/mL)	56.30 ± 32	46.3 ± 18	0.383‡‡	0.05*
ALP (U/L)	112 ± 27.7	78.7 ± 25	< 0.001*	< 0.001*
VitD (ng/mL)	38 ± 1§§	37.6 ± 14	0.935	0.676
CRP (mg/L) (Median and IQR)	4.5 (2.11–9.17)	2.64 (0.76–7.1)	0.16‡‡	-
BMD (g/cm^2^)	0.39 ± 0.2§§	0.42 ± 0.1¶¶	0.366	0.822
Smoking status (%)	33.3	30	-	-
Alcohol use (%)	43.3	20	-	-
CD4 count	485 ± 171†††	-	-	-

ALP, alkaline phosphatase; ART, antiretroviral treatment; BMD, bone mineral density; BMI, body mass index; CRP, C-reactive protein; CTx, C-terminal telopeptide; PTH, parathyroid hormone; SrCa, serum calcium; SrP, serum phosphate; TDF, tenofovir disoproxil fumarate.

† , *p*-value calculated from unpaired t-test;

‡ , *p*-value calculated from ANCOVA adjusted for smoking and alcohol use;

§ , Age already adjusted for with BMI when selecting the control group with propensity score matching;

¶ , *n* = 24 (4 participants unwilling to provide consent to access their medical records and missing information from 2 medical records);

†† , *n* = 22 (4 participants unwilling to provide consent to access their medical records and missing information from 4 medical files);

‡‡ , *p*-value calculated from Mann–Whitney test;

§§ , *n* = 25;

¶¶ , *n* = 18;

††† , *n* = 28; †††, *n* = 20.

* , *p* ≤ 0.05.

Regression analysis showed that HIV-infection status (TDF exposed) was significantly and independently associated with higher ALP (adjusted *r*^2^ = 0.310; *p* < 0.001).

The minimum and maximum plasma TFV concentration in HIV-infected women was 17.2 ng/mL and 434.2 ng/mL, respectively (Table 2). TFV plasma concentration was not detected in five participants hence their results were excluded from Table 3 analyses. The ART regimen consisted of TDF, lamivudine and efavirenz.

**TABLE 2:** Descriptive summary of tenofovir plasma concentrations.

Descriptive statistics and concentration categories	Tenofovir plasma concentration and number of samples
Mean (SD) ng/mL	114 ± 78.9
Median (IQR) ng/mL	113 (74–139)
Min/Max ng/mL	17.2/434.2
95% CI ng/mL	81.2–146.6
Undetected	*n* = 5
≤ 100 ng/mL	*n* = 12
100–120 ng/mL	*n* = 2
≥ 120 ng/mL	*n* = 11

IQR, interquartile range; SD, standard deviation.

**TABLE 3:** Comparisons of variables at tenofovir plasma concentrations of ≤100 ng/mL and ≥ 120 ng/mL in HIV-infected women.

Variable (mean and SD)	TFV plasma ≤100 ng/mL (*n* = 12)	TFV plasma ≥120 ng/mL (*n* = 11)	*p*
SrCa (mmol/L)	2.01 ± 0.19	2.12 ± 0.13	0.928
SrP (mmol/L)	1.01 ± 0.19	0.98 ± 0.15	0.11
CTx (ng/mL)	0.65 ± 0.31	0.77 ± 0.36	0.212
PTH (pg/mL)	56.14 ± 30.9	57.04 ± 26.4	0.037*
ALP (U/L)	105.43 ± 23.6	117.93 ± 29.8	0.151
VitD (ng/mL)	42.67 ± 4.2^a^	36.60 ± 8.6^b^	0.33
CRP (mg/L)	5.77 (2.77–9.03)^c^	3.82 (0.85–11.7)^c^	0.758
BMD (g/cm^2^)	0.39 ±0.13^d^	0.45 ± 0.17^e^	0.954

ALP, alkaline phosphatase; BMD, bone mineral density; CRP, C-reactive protein; CTx, C-terminal telopeptide; PTH, parathyroid hormone; SrCa, serum calcium; SrP, serum phosphate; TDF, tenofovir disoproxil fumarate; TFV, tenofovir.

a *n* = 8,

b *n* = 10,

c median(IQR),

d *n* = 9,

e *n* = 6.

* *p* ≤ 0.05.

Two participants had TFV concentration between 101 ng/mL and 119 ng/mL.

### Correlations between plasma tenofovir concentration, bone turnover markers and bone mineral density in HIV-infected women

There was no statistically significant correlation between plasma TFV concentration and SrCa (*r* = 0.371; *p* = 0.068), SrP (*r* = 0.189; *p* = 0.366), CTx (*r* = 0.135; *p* = 0.52), PTH (*r* = 0.162; *p* = 0.44), ALP (*r* = 0.234; *p* = 0.26), VitD (*r* = 0.006; *p* = 0.981) and BMD (*r* = 0.201; *p* = 0.472).

When TFV plasma concentration was used as conditioning variable, some significant correlations among BTM were observed at ≤ 100 ng/mL and ≥ 120 ng/mL of TFV plasma concentration. Hence, ≤ 100 ng/mL was the cut-off point for one group and ≥ 120 ng/mL for another.

### Comparisons between bone turnover markers and C-reactive protein at less than 100 ng/mL and above 120 ng/mL of tenofovir plasma concentration

There were no significant differences in SrCa, SrP, CTx, ALP, VitD, CRP and BMD (*p* > 0.05) between the two groups (≤ 100 ng/mL and ≥ 120 ng/mL). Nevertheless, PTH was higher (57.04 ± 26.4 vs. 56.14 ± 30.9; *p* = 0.037) at plasma TFV concentration ≥ 120 ng/mL compared to ≤ 100 ng/mL (Table 3).

### Correlations among serum calcium, parathyroid hormone, serum phosphate and C-terminal telopeptide in HIV-infected and uninfected women

There was no significant correlation between SrCa and PTH, SrCa and SrP or CTx and PTH in HIV-infected (TDF exposed) women [(Table 4), *p* > 0.05]. In HIV-uninfected women, a significant negative correlation was observed between SrCa and PTH (*r* = −0.49; *p* = 0.006), while significant positive correlations were observed between SrCa and SrP (*r* = 0.43; *p* = 0.019) as well as CTx and PTH (*r* = 0.56; *p* = 0.001), but no such associations were found in HIV-infected women.

**TABLE 4:** Correlations of serum calcium, parathyroid hormone, serum phosphate, C-terminal telopeptide and parathyroid hormone in HIV-infected and uninfected women.

Variables	HIV-infected (*n* = 30)		HIV-uninfected (*n* = 30)	
	Correlation coefficient (*r*)	*p*	Correlation coefficient (*r*)	*p*
SrCa and PTH	−0.19	0.305	−0.49	0.006*
SrCa and SrP	0.348	0.059	0.43	0.019*
CTx and PTH	0.33	0.072	0.56	0.001*

CTx, C-terminal telopeptide; PTH, parathyroid hormone; SrCa, serum calcium; SrP, serum phosphate.

* *p* ≤ 0.05.

## Discussion

In this pilot study, statistically significant higher ALP, CTx and PTH were observed in the HIV-infected women compared to the uninfected. This might be indicative of stimulated bone metabolism in HIV-infected women. A similar phenomenon was observed in the studies conducted by Stellbrink et al.,^5^ Samarawickrama et al.^8^ and Masiá et al.^21^ They reported an increase in PTH, ALP, BALP, P1NP and osteocalcin values in participants exposed to TDF treatment which was evidence of increased bone turnover.^5^ TDF-use was, however, not found to be associated with 25-dihydroxyvitamin D deficiency, hyperparathyroidism, increased BTM or reduced BMD.^8^

We did not find any significant correlation between TFV plasma concentration and BTM or BMD. Although there was this absence of the linear relationship, it did not imply that TFV does not have any role in bone metabolism. In order to fully evaluate the mean changes in BTM at different TFV plasma concentrations, Havens et al.^15^ compared BTM at ≤ 39.5 and > 95.3 ng/mL of TFV which represented the lowest 20% and highest 20% (quintile analysis) of the sample (*n* = 118), respectively. They found no mean differences in PTH, SrP, CTx and BALP but only in SrCa, free 1, 25-dihydroxyvitamin D and 25-dihydroxyvitamin D (*p* < 0.05). In the current study, there were no significant mean differences in CTx, ALP, SrP, VitD or absolute BMD at ≤ 100 ng/mL and ≥ 120 ng/mL which represented approximately the lower half and upper half of the sample (*n* = 25), respectively. Meanwhile, there was a trend of higher ALP and CTx concentrations, although not significant, at plasma TFV ≥ 120 ng/mL, while VitD and SrP tended to be lower. The mean PTH was, however, significantly higher at ≥ 120 ng/mL plasma TFV which suggested the possibility of stimulated bone resorption, as higher plasma PTH concentrations are associated with low BMD.^22^ This could also be as a result of TFV-induced cellular stress that affects osteoblast function leading to decreased bone formation as proposed by Grigsby et al.^13^ In the study by Gervasoni et al. (*n* = 133),^23^ higher TFV plasma concentrations (288 ng/mL ± 173 ng/mL) were found in patients with altered osteocalcin (bone formation marker) concentrations than in patients with normal osteocalcin concentrations (153 ng/mL ± 115 ng/mL, *p* < 0.01).

In the previous published data of the same participants, there was no indication of deterioration in renal function in HIV-infected participants.^17^ In fact, both estimated creatinine clearance (112.5 ± 40.5 mL/min vs. 104.9 ± 43 mL/min, *p* = 0.048) and estimated glomerular filtration rate (125.8 ± 39.6 mL/min vs. 105 ± 25.5 mL/min/1.73 m^2^, *p* = 0.047) were significantly higher in HIV-infected participants compared to the uninfected control group.^17^ Therefore, the influence of deterioration in renal function on serum concentration of BTMs that are cleared by the kidney could not be assumed in our study.

In the current study, the influence of inflammation on bone metabolism was not evident even though CRP concentrations in HIV-infected women tended to be higher than in uninfected controls (*p* = 0.16) and no correlation was found between TFV concentration, BTM and BMD. Our finding agrees in part with Gervasoni et al.^23^ who did not find the association between plasma TFV concentration and CTx. Similarly, in a study performed by Havens et al.^15^ of mostly African American race (*n* = 118), no correlation was found between plasma TFV concentration and SrCa, PTH, SrP, BALP and CTx. The authors, however, reported a negative correlation between plasma TFV concentrations and free 1,25-dihydroxyvitamin D.^15^ Total VitD (25-dihydroxyvitamin D) was measured in this study and not just 1,25-dihydroxyvitamin D as in the study of Havens et al.^15^

Higher bone turnover was reported in patients on TDF containing regimens. CTx, P1NP and BALP were higher in populations of mostly Caucasian males (*n* = 154, *n* = 193) than those exposed to non-TDF containing regimens.^5,7^ Exposure to TDF containing regimens was associated with increased total ALP that indicated osteoblast activation.^24,25^ Total ALP had been used to monitor bone metabolism and showed high correlation with bone-specific BALP. The increase in total ALP in the current study was most likely contributed by BALP as no cholestatic side effects of TDF have been described.^24^

In a cross-sectional study of women (26% African American) aged above 50 years (*n* = 31), mean BMD was significantly lower in the HIV-infected ART exposed than uninfected control group at the lumbar spine and total hip.^26^ In the current study, BMD of the distal and ultra-distal sites of the forearm in both HIV-infected TDF-exposed and uninfected controls was similar. This difference in results may be because of the fact that in the former study,^26^ BMDs were not adjusted for confounding effects of smoking status and alcohol consumption unlike in our study. Dave et al.^27^ determined factors associated with low BMD in HIV-infected South Africans. They reported that exposure to efavirenz or lopinavir-based ART was associated with lower total hip BMD, whereas higher body weight and higher serum VitD concentration were associated with higher total hip BMD. The use of efavirenz-based ART was also independently associated with lumbar spine BMD.^27^ Bedimo et al.^28^ found that HIV-infection and hepatitis C virus infection were significantly associated with low BMD but when the statistical models were adjusted for TDF-use, it greatly diminished the association. Their findings suggested that the effect of HIV-infection on BMD was principally driven by TDF.

Bone remodelling is a constant process tightly coupled to bone resorption and bone formation mediated by osteoclasts and osteoblasts, respectively.^6^ Bone formation and resorption markers are well correlated to one another; thus, a change in bone turnover signals a disturbance in well-controlled bone remodelling processes.^6,29^ Furthermore, there is regulated interplay among bone mineral metabolic parameters, namely PTH, SrCa and SrP, in the bone remodelling process.^30,31^ In the current study, we found significant correlations between SrCa and PTH, SrCa and SrP as well as CTx and PTH in the HIV-uninfected participants who indicated expected synchronisation of bone mineral metabolic processes. The correlations in the HIV-infected group were in the same direction as uninfected but no statistically significant correlations were observed between SrCa and PTH, SrCa and SrP or CTx and PTH in HIV-infected participants. This may be because of the disturbance in the regulation process of bone metabolic parameters likely attributed to ART and HIV-infection status.^21,32^ The lack of significant correlation may also be because of larger within group variation in PTH in the HIV-infected compared with uninfected group (Table 1).

### Limitations of the study

This study involves an under-investigated population in a resource-limited setting, but has some limitations to consider: the cross-sectional nature of the study rules out assessments of causal relationship between plasma TFV concentration and BTM. The small number of participants does not allow generalising the findings although significant associations and trends observed need to be confirmed in longitudinal studies with larger sample sizes. The influence of TDF exposure time, VitD deficiency and sufficiency as categorisation could not be assessed as it was not statistically sensible owing to a small sample size. Nevertheless, availability of a control group to make comparisons added strength to this study. No TFV was detected in five participants and they were therefore excluded in the comparison analyses that involved TFV concentrations. However, the effect of TFV on bone density is long term and these participants were on a TDF-based regimen for an average duration of 16 months. These findings stimulate the interest of further research into plasma TFV concentrations and bone metabolism in black women for early detection of bone loss.

## Conclusion

In conclusion, the observed increased PTH concentration and a higher trend in CTx and SrCa at ≥ 120 ng/mL suggest the possibility of increased rate of bone turnover at higher TFV plasma concentrations within this study. Additionally, there were perturbations in bone turnover process in HIV-infected participants (TFV-exposed) compared to the HIV-uninfected controls. Further studies with larger sample size are needed to confirm these results.

## References

[1] Powderly WG. Osteoporosis and bone health in HIV. Curr HIV/AIDS Rep. 2012;9(3):218–222. 10.1007/s11904-012-0119-7 22581359

[2] Schafer JJ, Manlangit K, Squires KE. Bone health and human immunodeficiency virus infection. Pharmacotherapy. 2013;33(6):665–682. 10.1002/phar.1257 23553497

[3] Hansen A, Obel N, Nielsen H, Pedersen C, Gerstoft J. Bone mineral density changes in protease inhibitor-sparing vs. nucleoside reverse transcriptase inhibitor-sparing highly active antiretroviral therapy: Data from a randomized trial*. HIV Med. 2011;12(3):157–165. 10.1111/j.1468-1293.2010.00864.x 20722752

[4] Parsonage M, Wilkins E, Snowden N, Issa B, Savage M. The development of hypophosphataemic osteomalacia with myopathy in two patients with HIV infection receiving tenofovir therapy. HIV Med. 2005;6(5):341–346. 10.1111/j.1468-1293.2005.00318.x 16156882

[5] Stellbrink H, Orkin C, Arribas JR, et al. Comparison of changes in bone density and turnover with abacavir-lamivudine versus tenofovir-emtricitabine in HIV-infected adults: 48-week results from the ASSERT study. Clin Infect Dis. 2010;51(8):963–972. 10.1086/656417 20828304

[6] Haskelberg H, Carr A, Emery S. Bone turnover markers in HIV disease. AIDS Rev. 2011;13:240–250. 21975360

[7] Haskelberg H, Hoy JF, Amin J, Ebeling PR, Emery S, Carr A. Changes in bone turnover and bone loss in HIV-infected patients changing treatment to tenofovir-emtricitabine or abacavir-lamivudine. PLoS One. 2012;7(6):e38377. 10.1371/journal.pone.0038377 22719882 PMC3376146

[8] Samarawickrama A, Jose S, Sabin C, Walker-Bone K, Fisher M, Gilleece Y. No association between vitamin D deficiency and parathyroid hormone, bone density and bone turnover in a large cohort of HIV-infected men on tenofovir. J Int AIDS Soc. 2014;17(4):19568. 10.7448/IAS.17.4.19568 25394075 PMC4224824

[9] Childs K, Welz T, Samarawickrama A, Post FA. Effects of vitamin D deficiency and combination antiretroviral therapy on bone in HIV-positive patients. AIDS. 2012;26(3):253–262. 10.1097/QAD.0b013e32834f324b 22112601

[10] Childs KE, Fishman SL, Constable C, et al. Inadequate vitamin D exacerbates parathyroid hormone elevations in tenofovir users. AIDS Res Hum Retroviruses. 2010;26(8):855–859. 10.1089/aid.2009.0308 20672993 PMC2957627

[11] Klassen K, Martineau AR, Wilkinson RJ, Cooke G, Courtney AP, Hickson M. The effect of tenofovir on vitamin D metabolism in HIV-infected adults is dependent on sex and ethnicity. PLoS One. 2012;7(9):e44845. 10.1371/journal.pone.0044845 22984574 PMC3440360

[12] Rosenvinge MM, Gedela K, Copas AJ, et al. Tenofovir-linked hyperparathyroidism is independently associated with the presence of vitamin D deficiency. J Acquir Immune Defic Syndr. 2010;54(5):496–499. 10.1097/QAI.0b013e3181caebaa 20672448

[13] Grigsby IF, Pham L, Mansky LM, Gopalakrishnan R, Mansky KC. Tenofovir-associated bone density loss. Ther Clin Risk Manag. 2010;6:41–47. 10.2147/TCRM.S8836 20169035 PMC2817787

[14] Kearney BP, Flaherty JF, Shah J. Tenofovir disoproxil fumarate: Clinical pharmacology and pharmacokinetics. Clin Pharmacokinet. 2004;43(9):595–612. 10.2165/00003088-200443090-00003 15217303

[15] Havens PL, Kiser JJ, Stephensen CB, et al. Association of higher plasma vitamin d binding protein and lower free calcitriol levels with tenofovir disoproxil fumarate use and plasma and intracellular tenofovir pharmacokinetics: Cause of a functional Vitamin D deficiency? Antimicrob Agents Chemother. 2013;57(11):5619–5628. 10.1128/AAC.01096-13 24002093 PMC3811269

[16] Teo K, Chow CK, Vaz M, Rangarajan S, Yusuf S. The Prospective Urban Rural Epidemiology (PURE) study: Examining the impact of societal influences on chronic noncommunicable diseases in low-, middle-, and high-income countries. Am Heart J. 2009;158(1):1–7. 10.1016/j.ahj.2009.04.019 19540385

[17] Mulubwa M, Rheeders M, Fourie C, Viljoen M. Associations between plasma tenofovir concentration and renal function markers in HIV-infected women. S Afr J HIV Med. 2016;17(1):a458. 10.4102/sajhivmed.v17i1.458 PMC5843126 29568614

[18] Mulubwa M, Rheeders M, Du Plessis L, Grobler A, Viljoen M. Development and validation of high performance liquid chromatography tandem mass spectrometry (HPLC-MS/MS) method for determination of tenofovir in small volumes of human plasma. J Chromatogr Sep Tech. 2015;6(7):300. 10.4172/2157-7064.1000300

[19] Rodríguez-Nóvoa S, Labarga P, D’Avolio A, et al. Impairment in kidney tubular function in patients receiving tenofovir is associated with higher tenofovir plasma concentrations. AIDS. 2010;24(7):1064–1066. 10.1097/QAD.0b013e32833202e2 20299966

[20] Kruger IM, Kruger MC, Doak CM, Kruger A. Cut-off values of distal forearm bone density for the diagnosis of central osteoporosis in black postmenopausal South African women. JEMDSA. 2012;17(2):78–83. 10.1080/22201009.2012.10872281

[21] Masiá M, Padilla S, Robledano C, López N, Ramos JM, Gutiérrez F. Early changes in parathyroid hormone concentrations in HIV-infected patients initiating antiretroviral therapy with tenofovir. AIDS Res Hum Retroviruses. 2012;28(3):242–246. 10.1089/AID.2011.0052 21639815

[22] Pocaterra D, Carenzi L, Ricci E, et al. Secondary hyperparathyroidism in HIV patients: Is there any responsibility of highly active antiretroviral therapy? AIDS. 2011;25(11):1430–1433. 10.1097/QAD.0b013e328349060e 21617518

[23] Gervasoni C, Minisci D, Meraviglia P, et al. Determinants of bone diseases in tenofovir-treated HIV patients. AIDS. 2016;30(10):1686–1687. 10.1097/QAD.0000000000001094 27243779

[24] Fux CA, Rauch A, Simcock M, et al. Tenofovir use is associated with an increase in serum alkaline phosphatase in the Swiss HIV Cohort Study. Antivir Ther. 2008;13:1077–1082. 19195333

[25] Mueller NJ, Fux CA, Ledergerber B, et al. High prevalence of severe vitamin D deficiency in combined antiretroviral therapy-naive and successfully treated Swiss HIV patients. AIDS. 2010;24(8):1127–1134. 10.1097/QAD.0b013e328337b161 20168200

[26] Yin M, Dobkin J, Brudney K, et al. Bone mass and mineral metabolism in HIV postmenopausal women. Osteoporosis Int. 2005;16(11):1345–1352. 10.1007/s00198-005-1845-0 PMC3108020 15754081

[27] Dave JA, Cohen K, Micklesfield LK, Maartens G, Levitt NS. Antiretroviral therapy, especially efavirenz, is associated with low bone mineral density in HIV-infected South Africans. PLoS One. 2015;10(12):e0144286. 10.1371/journal.pone.0144286 26633015 PMC4669137

[28] Bedimo R, Cutrell J, Zhang S, et al. Mechanisms of bone disease in HIV and hepatitis C virus: Impact of bone turnover, tenofovir exposure, sex steroids and severity of liver disease. AIDS. 2016;30(4):601–608. 10.1097/QAD.0000000000000952 26558726

[29] Nomura Y, Yoshizaki A, Yoshikata H, et al. Study of the distribution by age group of serum cross-linked C-terminal telopeptide of type I collagen and procollagen type I N-propeptide in healthy Japanese women to establish reference values. J Bone Miner Metab. 2013;31(6):644–651. 10.1007/s00774-013-0460-y 23579312

[30] Dhingra R, Sullivan LM, Fox CS, et al. Relations of serum phosphorus and calcium levels to the incidence of cardiovascular disease in the community. Arch Intern Med. 2007;167(9):879–885. 10.1001/archinte.167.9.879 17502528

[31] Jayasena CN, Modi M, Palazzo F, et al. Associations of serum 25-hydroxyvitamin D with circulating PTH, phosphate and calcium in patients with primary hyperparathyroidism. Clin Endocrinol. 2013;78(6):838–843. 10.1111/cen.12062 23036072

[32] Gutiérrez F, Masiá M. The role of HIV and antiretroviral therapy in bone disease. AIDS Rev. 2011;13(2):109–118. 21587342

